# Predictive Added Value of Selected Plasma Lipids to a Re-estimated Minimal Risk Tool

**DOI:** 10.3389/fcvm.2021.682785

**Published:** 2021-07-16

**Authors:** Antonella Bodini, Elena Michelucci, Nicoletta Di Giorgi, Chiara Caselli, Giovanni Signore, Danilo Neglia, Jeff M. Smit, Arthur J.H.A. Scholte, Pierpaolo Mincarone, Carlo G. Leo, Gualtiero Pelosi, Silvia Rocchiccioli

**Affiliations:** ^1^Institute for Applied Mathematics and Information Technologies “E. Magenes,” National Research Council, Milan, Italy; ^2^Institute of Clinical Physiology, National Research Council, Pisa, Italy; ^3^Cardiovascular Department, Fondazione Toscana G. Monasterio, Pisa, Italy; ^4^NEST, Scuola Normale Superiore, Pisa, Italy; ^5^Fondazione Pisana per la Scienza, San Giuliano Terme, Italy; ^6^Department of Cardiology, Leiden University Medical Centre, Leiden, Netherlands; ^7^Institute for Research on Population and Social Policies, National Research Council, Brindisi, Italy; ^8^Institute of Clinical Physiology, National Research Council, Lecce, Italy

**Keywords:** biomarkers evaluation, coronary artery disease, lipidomics, coronary computed tomography angiography, likelihood ratio test, pre-test clinical models

## Abstract

**Background:** Lipidomics is emerging for biomarker discovery in cardiovascular disease, and circulating lipids are increasingly incorporated in risk models to predict cardiovascular events. Moreover, specific classes of lipids, such as sphingomyelins, ceramides, and triglycerides, have been related to coronary artery disease (CAD) severity and plaque characteristics. To avoid unnecessary testing, it is important to identify individuals at low CAD risk. The only pretest model available so far to rule out the presence of coronary atherosclerosis in patients with chest pain, but normal coronary arteries, is the minimal risk tool (MRT).

**Aim:** Using state-of-the-art statistical methods, we aim to verify the additive predictive value of a set of lipids, derived from targeted plasma lipidomics of suspected CAD patients, to a re-estimated version of the MRT for ruling out the presence of coronary atherosclerosis assessed by coronary CT angiography (CCTA).

**Methods:** Two hundred and fifty-six subjects with suspected stable CAD recruited from five European countries within H2020-SMARTool, undergoing CCTA and blood sampling for clinical biochemistry and lipidomics, were selected. The MRT was validated by regression methods and then re-estimated (reMRT). The reMRT was used as a baseline model in a likelihood ratio test approach to assess the added predictive value of each lipid from 13 among ceramides, triglycerides, and sphingomyelins. Except for one lipid, the analysis was carried out on more than 240 subjects for each lipid. A sensitivity analysis was carried out by considering two alternative models developed on the cohort as baseline models.

**Results:** In 205 subjects, coronary atherosclerosis ranged from minimal lesions to overt obstructive CAD, while in 51 subjects (19.9%) the coronary arteries were intact. Four triglycerides and seven sphingomyelins were significantly (*p* < 0.05) and differentially expressed in the two groups and, at a lesser extent, one ceramide (*p* = 0.067). The probability of being at minimal risk was significantly better estimated by adding either Cer(d18:1/16:0) (*p* = 0.01), SM(40:2) (*p* = 0.04), or SM(41:1) at a lesser extent (*p* = 0.052) to reMRT than by applying the reMRT alone. The sensitivity analysis confirmed the relevance of these lipids. Furthermore, the addition of SM(34:1), SM(38:2), SM(41:2), and SM(42:4) improved the predictive performance of at least one of the other baseline models. None of the selected triglycerides was found to provide an added value.

**Conclusions:** Plasma lipidomics can be a promising source of diagnostic and prognostic biomarkers in cardiovascular disease, exploitable not only to assess the risk of adverse events but also to identify subjects without coronary atherosclerosis, thus reducing unnecessary further testing in normal subjects.

## Introduction

The latest European Society of Cardiology (ESC) guidelines for the diagnosis and management of chronic coronary syndromes (1) recommend coronary CT angiography (CCTA) as the first-line diagnostic test for patients with low–intermediate clinical likelihood of obstructive coronary artery disease (CAD). One of the novelties of the latest ESC guidelines is the concept of clinical likelihood of CAD, which utilizes several conventional risk factors of CAD as pretest probability modifiers (1, 2). Among the existing validated pretest probability (PTP) models of obstructive CAD, a few can be used to rule out CAD (1, 3). The minimal risk tool (MRT) model has been recently introduced as a novel PTP model using only conventional variables measured in clinical practice to identify patients with chest pain, normal coronary arteries, and no adverse events at follow-up who derive minimal benefit and value from first-line diagnostic tests of CAD such as CCTA (4). The performance of MRT has been tested in different populations (5, 6), and an updated model has been proposed (6). Furthermore, thanks to the current advancements in analytical technologies, many additional metabolites and lipids can be easily measured in plasma samples and tested as candidate biomarkers of cardiometabolic risk. Metabolomics and lipidomics are promising sources of novel biomarkers of cardiovascular disease (7): in particular, specific plasma lipid species have been suggested to play a role in the pathogenesis of atherosclerosis (8–11), metabolic disorders, and clinical manifestations of cardiovascular disease in general (12). In addition, we have recently demonstrated that, even under optimal cholesterol-lowering treatments, CAD severity and atherosclerotic non-calcific plaque burden are significantly associated with specific circulating lipid species among the classes of sphingomyelins (SMs) and phosphatidylethanolamines (PEs), supporting their clinical exploitation as biomarkers of obstructive CAD with potentially vulnerable lesions (13).

In agreement with Hlatky et al. (14) and Moons et al. (15), a new-generation biomarker should not only have a diagnostic value *per se*, i.e., a statistically significant association with the outcome, but it should also provide a significant added value to the improvement of risk estimation when combined with other risk markers. Recently, for instance, the discrimination improvement of the polygenic risk scores with respect to the pooled cohort risk equations was called into question by statistical analyses carried out on large populations (16).

In this proof-of-concept study, we evaluated the value of lipidomics-derived markers in the existing pretest for the diagnostic workup of CAD patients using state-of-the-art statistical methods (17). We first carried out a full validation of the MRT on a population with a relatively low prevalence of subjects at minimal risk. Then, for the first time, in stable CAD patients (H2020-689068-SMARTool Project clinical trial, ClinicalTrials.gov identifier: NCT04448691), we evaluated whether and which plasma lipids can significantly contribute to improve the identification of subjects at minimal risk. Finally, a sensitivity analysis was conducted by considering also their added value to two alternative models predicting minimal risk inspired by PTP models of CAD.

## Materials and Methods

### Patient Population

The study population consists of 256 subjects from the clinical trial of the SMARTool Project (H2020-689068-SMARTool Project clinical trial, Clinicaltrial.gov identifier: NCT04448691). Subjects with suspected coronary artery disease and a median pretest probability of obstructive CAD of 65% (interquartile range, 33–75%)—intermediate risk (18)—were recruited in seven clinical centers from five European countries (Finland, Italy, Poland, Spain, and Switzerland). Clinical data, risk factors, clinical biochemistry, and stored plasma samples were retrospectively collected. Coronary atherosclerosis was assessed by CCTA, and plasma samples were used for standard clinical biochemistry and lipidomics analysis. Previous cardiac surgery, coronary revascularization, or major adverse cardiovascular events in the last 6 months, chronic kidney disease and atrial fibrillation were the main exclusion criteria.

### Clinical and Laboratory Data

All variables included in the MRT were evaluated for this analysis, except for symptoms of physical/mental stress, unknown for all subjects, and ethnicity, the entire population being Caucasian. Hypertension was defined as a blood pressure >140/90 mmHg on at least two occasions (>130/80 mmHg for patients with diabetes) or requiring antihypertensive treatment. Diabetes was defined as a history of diabetes, an elevated fasting serum glucose >126 mg/dl (7 mmol/l), or antidiabetic therapy. Dyslipidemia was defined as an elevated cholesterol level [total cholesterol >200 mg/dl (5.18 mmol/l), low-density lipoprotein >130 mg/dl (3.37 mmol/l), or high-density lipoprotein <40 mg/dl (1.04 mmol/l) in men and <50 mg/dl (1.30 mmol/l) in women] or treatment with cholesterol-lowering medications. Smoking history was defined as current or past smoking. Plasma concentrations of cardiac troponin T were measured using the high-sensitive method on COBAS E411 with Elecsys Troponin T-hs STAT by Roche Diagnostics (Basel, Switzerland) (19).

### Plasma Lipid Measurements

The plasma samples of all patients were stored in the IFC Biobank. For lipid extraction, they were thawed at room temperature and immediately processed. Total lipid extraction from an aliquot of plasma was performed according to the procedure of Folch et al. (20). The specific quantitative procedure used for HPLC-MS/MS analysis is reported in Michelucci et al. (13).

### Coronary CCTA Analysis

Coronary CCTA was performed according to a predefined standard operating procedure to ensure optimal image quality. All images were analyzed blinded to clinical data by a separate Core Laboratory (Leiden University Medical Center) by two independent cardioradiologists. The coronary arteries were assessed according to the modified 17-segment American Heart Association classification (21) and the Coronary Artery Disease Reporting and Data System (CAD-RADS) guidelines (22). Patients were defined as either normal or with CAD according to the following criteria: normality was defined as no evidence of coronary atherosclerosis at CCTA scan, a coronary calcium score of 0, and no previous cardiovascular events at clinical history; the presence of CAD was assessed by CCTA (CAD-RADS classes 1–5) and/or a calcium score >0. All normal subjects were also free of adverse events in the following 4–8 years, in agreement with (4).

### Descriptive Statistical Analysis

Continuous variables were described using means and standard deviations (μ ± σ), or medians (μ_0.5_) and interquartile ranges (IQRs, reported as the lower–upper quartile interval) when data distributions showed a marked lack of symmetry and/or the presence of several suspected outliers. Categorical variables were described using frequencies and percentages. Group comparisons with respect to continuous variables were performed using either the *t*-test or the Mann–Whitney test, when graphical indications of non-normality existed; Pearson's chi-square test or Fisher's exact test was used for comparisons with respect to categorical variables. Two-tailed tests were considered, and the significance level was set at α = 0.05. All analyses were performed using R Statistical Software (R Project for Statistical Computing, RRID:SCR_001905) (23).

Missing data imputation has been applied with a threshold of <1% to ensure that the analysis and its results were not significantly guided by the imputation method selected. The mean or the median has been considered each time as the imputed value, and the gender of the subject has also been taken into account when the distribution of the variable in the genders differed significantly.

### MRT Validation

Calibration of the MRT model on our cohort was visually checked by plotting the predicted vs. the observed risk within each decile of risk, as estimated by the MRT, and then tested for significance with the Hosmer–Lemeshow (HL) statistic, as usual. The validation analysis was then deepened by testing calibration in-the-large, as well as both the overall and specific effects of the predictors by logistic regression techniques (24), even by re-estimating the model, if necessary.

### LRT Analysis

The capability of each lipid statistically associated with the outcome to improve the prediction of subjects at minimal risk was evaluated by a likelihood ratio test (LRT) between the (possibly re-estimated) MRT as the baseline model and an enhanced model obtained by adding the lipid to the baseline model (25, 26). To apply the test, we firstly computed the logit transformation, *x*_*i*_, of the risks predicted by the baseline model (i.e., *x*_*i*_ is the linear combination obtained from the estimated parameters and the selected risk factors in the baseline model). Then, the LRT evaluates the incremental value brought by any lipid to the estimation of the binary outcome of being at minimal risk, *y*_*i*_, by comparing the likelihood of the logistic model corresponding to the baseline linear combination (univariable, *y*_*i*_ = α_0_ + α_1_*x*_*i*_) to the likelihood of the enhanced linear combination (bivariable, *y*_*i*_ = β_0_+β_1_*l*_*i*_+β_2_*x*_*i*_) obtained by adding (on top) the lipid of interest, *l*_*i*_, to the baseline linear prediction risk. The calibration in-the-large of the baseline model on the subset of subjects defined by the considered lipid was checked and the goodness-of-fit verified by the Hosmer–Lemeshow test, each time. Log transformations of lipids were also considered for skewed distributions. The LRT was carried out by using the R package “lmtest” (27).

A possibly re-estimated minimal risk tool is our main baseline model. To carry out a sensitivity analysis on the added value of the considered set of lipids, two other baseline models based on current literature have been developed on the SMARTool cohort. Firstly, a multivariable logistic regression model including age, sex, and typicality of chest pain (basic model) has been estimated. The choice of predictors is clearly inspired by the Diamond–Forrester model and by the ESC guidelines, commonly used as the basis for any comparison when CAD is addressed. Of course, the basic model is a completely new model due to the minimal risk endpoint here considered. Secondly, a multivariable logistic regression model including age, sex, typicality of chest pain, and high-sensitive cardiac troponin T (basic-hs-cTnT model) has been considered as per the recently recognized role of high-sensitive troponin as an independent predictor of coronary artery disease (28–31). The validity of these two models as baseline models was checked by the Hosmer–Lemeshow test and *c-*statistics [area under the receiver operating characteristic (ROC) curve (AUC) value].

Finally, a multivariable logistic regression model was also fitted using the backward variable selection method with all the lipids that resulted in a significantly incremental predictive value for at least one of the considered baseline models. The final combination of lipids was in turn tested for improvement by the LRT approach.

## Results

### Study Population

The cohort consisted of 96 women (37.5%) and 160 men; the mean age was 62.4 (±8.1) years, and 16 subjects (6.3%, four women) were <50 years old. The mean age was not significantly different between the two sexes (63.3 vs. 61.8 years, *p* = 0.14). Only statins were used as lipid-lowering therapies, and 52.3% of subjects were on statins (52.1% of women and 52.5% of men). The demographic and clinical characteristics of the 256 subjects are listed in Table 1 according to their classification in the two groups. The summary values do not account for imputation. Subjects defined as normal (51, 19.9%) were mostly females (72.5 vs. 28.8%, *p* ≪ 0.001), were significantly younger (mean = 58.4 vs. 63.4 years, *p* ≪ 0.001), and had a lower prevalence of hypertension (52.9 vs. 70.7%, *p* = 0.024) and diabetes (9.8 vs. 24.9%, *p* = 0.032) than the CAD patients. They also had significantly higher high-density lipoprotein cholesterol (HDL-C; median = 56.0 mg/dl, IQR = 47.5–62.5 mg/dl) than the other subjects (median = 50.0 mg/dl, IQR = 41.0–59.0 mg/dl, *p* = 0.016). These results are in line with the findings obtained both in the derivation cohort by Fordyce et al. (4) and in the Dan-NICAD validation cohort (6). Moreover, significantly fewer subjects at minimal risk used angiotensin-converting enzyme (ACE) inhibitors/angiotensin receptor blocker (ARBs) (*p* = 0.006). The difference in the use of either statins or aspirin was only weakly significant (*p* < 0.065).

**Table 1:** Selected demographic and clinical characteristics.

**Clinical characteristics^**a**^**	**Normal (*n* = 51)**	**CAD (*n* = 205)**	***p*-value^**b**^**
**Demography**			
Age (years), μ ± σ	58.4 ± 7.35	63.4 ± 7.93	≪0.001
Female sex	72.5%	28.8%	≪0.001
**Clinical factors**			
BMI	27.3 ± 3.53 [49]	27.6 ± 3.80 [202]	0.64
Current smoker	9.8%	17.6%	0.255
Hypertension	52.9%	70.7%	0.024
Diabetes	9.8%	24.9%	0.032
Dyslipidemia	84.3%	86.3% [204]	0.893
Family history of CAD	49.0%	43.4%	0.573
**Clinical symptoms**			
Typical chest pain	35.3%	18.0%	0.003
Atypical chest pain	49.0%	44.9%	
Non-anginal chest pain	15.7%	37.1%	
**Biomolecular characterization**			
Triglycerides (mg/dl), μ_0.5_ (IQR)	89 (70–124)	112.5 (81–158.3) [204]	0.007
Total cholesterol (mg/dl), μ_0.5_ (IQR)	199 (161–231)	181 (146–212)	0.045
HDL-C (mg/dl), μ_0.5_ (IQR)	56.0 (47.5–62.5)	50.0 (41–59) [204]	0.016
Hs-cTnT (ng/L), μ_0.5_ (IQR)	3.59 (3.00–4.96)[45]	6.99 (4.43–9.32) [180]	≪0.001
**Therapies**			
Statins	39.2%	55.6%	0.052
ACE inhibitors/ARBs	31.4%	54.1%	0.006
Diuretics	11.8%	19.0%	0.31
β-blockers	47.1%	43.9%	0.80
Aspirin	49.0%	64.4%	0.063

*Summaries are derived on available data and do not account for imputations*.

a *For categorical variables, data are presented as percentages. Pearson's chi-square test or Fisher's exact test was used for comparisons. For continuous variables, either the mean and standard deviation, μ ± σ, or the median and interquartile range expressed as an interval, μ_0.5_ (IQR), is specified from time to time, according to either normality or non-normality graphical indications. The test is either the two-sided t-test or the Mann–Whitney test, respectively. The number of subjects with available data is reported in square brackets if different from the total number in the group (normal and CAD)*.

b *p ≪ 0.001 means order of magnitude <-4*.

While traditional risk factors and most of the lipids had at most 0.1% missing data, a stronger presence of missing data was found for high-sensitive cardiac troponin T (hs-cTnT), Cer(d18:1/18:0), SM(34:1), SM(40:3), and SM(40:1). In the former case, we imputed the missing data to either the mean/median or the prevalent category. In the latter, no imputation technique was applied.

Table 2 shows the summary statistics of the distributions of the 20 selected lipids according to the two groups of normal and CAD subjects. All but Cer(d18:1/18:0), TG(50:1), TG(50:2), SM(40:1), and SM(42:1) were proven to have strongly significantly different distributions in the two groups, while Cer(d18:1/16:0) showed only a weakly significant difference (*p* = 0.067). In particular, the triglyceride (TG) levels are lower in the normal group while SMs are higher, as well as Cer(d18:1/16:0). All the lipids with significant *p*-values were considered for improvement testing. Additionally, despite its weaker significance, Cer(d18:1/16:0) was retained due to its established relevance in the assessment of the severity of stenosis (32).

**Table 2:** Considered ceramides, triglycerides, and sphingomyelins.

**Lipid species**	**Normal (μmol/L)^**a**^** **(*n* = 51)**	**CAD (μmol/L)^**a**^** **(*n* = 205)**	***p*-value^***b***^**
Cer(d18:1/16:0)	0.52 (0.31–0.75)	0.43 (0.29–0.62)	0.067
Cer(d18:1/18:0)	0.09 (0.06–0.11) [46]	0.09 (0.06–0.14) [181]	0.61
TG(50:1)	185.5 (108.5–235.5)	198.2 (144.4–269.9)	0.12
TG(50:2)	66.1 (42.8–109.9)	81.7 (55.9–116.5)	0.09
TG(52:2)	182.6 (158.5–219.1)	205.8 (175.5–853.7)	0.008
TG(52:3)	74.1 (62.0–99.4)	92.8 (73.0–112.4)	0.004
TG(54:2)	50.8 (31.5–63.4)	56.2 (40.3–85.32)	0.027
TG(54:3)	72.1 (51.8–86.6)	82.2 (59.7–99.0)	0.016
SM(34:1)	139.7 (131.6–151.4) [46]	134.3 (121.8–143.7) [180]	0.013
SM(36:2)	24.4 (19.9–27.7)	20.9 (17.6–24.7)	0.002
SM(38:2)	14.0 (12.1–16.2)	12.4 (10.3–14.7)	0.001
SM(38:1)	49.0 (42.6–54.99)	44.6 (37.6–54.0)	0.078
SM(40:3)	7.3 (6.4–9.2) [50]	6.6 (5.3–9.0) [191]	0.046
SM(40:2)	69.4 (61.8–81.2)	61.2 (52.2–73.2)	0.002
SM(40:1)	114.9 (94.2–124.6) [48]	107.1 (92.5–122.5) [195]	0.19
SM(41:2)	49.9 (40.7–60.3)	41.8 (33.8–52.3)	0.003
SM(41:1)	58.9 (49.3–72.2)	52.0 (42.4–68.4)	0.035
SM(42:4)	7.6 (6.4–10.1)	6.8 (5.6–9.2)	0.030
SM(42:3)	99.9 (89.7–115.2) [50]	94.7 (77.9–109.1) [204]	0.024
SM(42:1)	87.7 (67.0–100.0)	79.0 (65.3–97.5) [203]	0.15

*Summaries are derived on available data and do not account for imputations*.

a *Median and interquartile range expressed as an interval, μ_0.5_ (IQR)*.

b *The test is the two-sided Mann–Whitney test. The number of subjects with available data is reported in square brackets if different from the total number in any of the two groups (normal and CAD)*.

### MRT Validation and Re-Estimation

**Figure 1:**
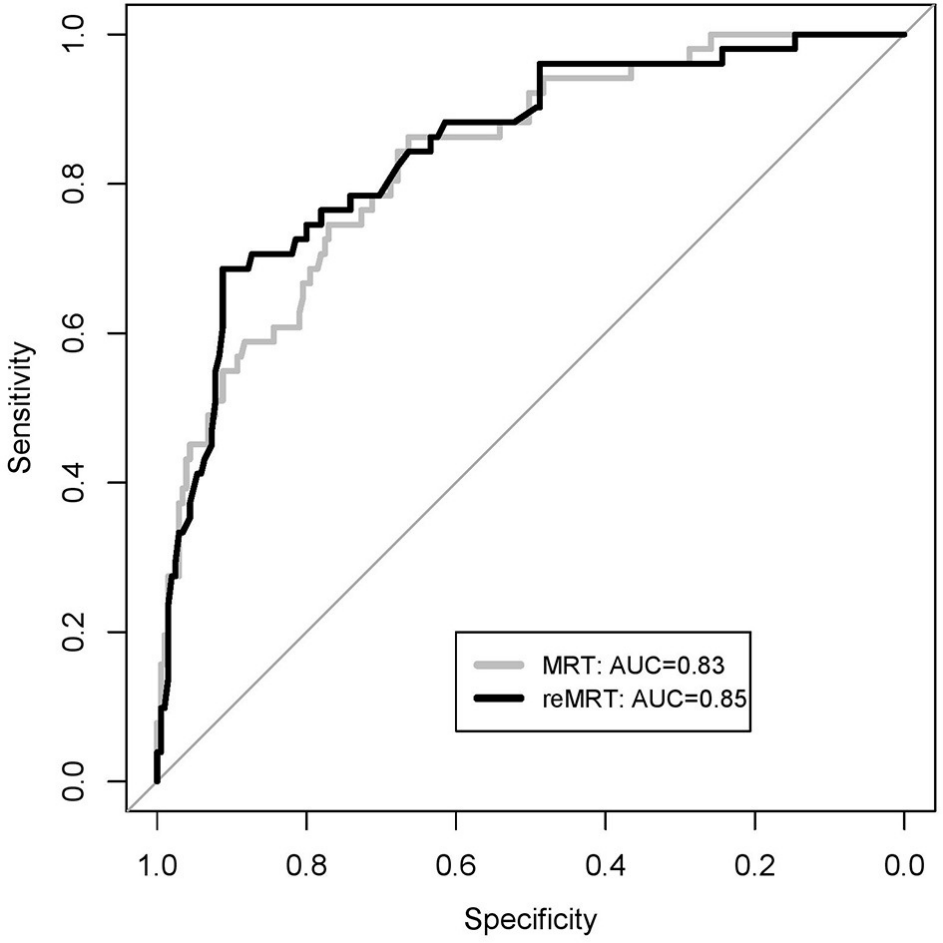
Receiver operating characteristic (ROC) curves of the minimal risk tool (MRT) model and the re-estimated MRT model (reMRT) on the SMARTOOL cohort.

The probability of minimal risk in the SMARTool cohort was firstly computed using the published MRT coefficients (4). Regression assessment of calibration-in-the-large did not show a significant difference between the mean observed outcome and the MRT predicted probability (19.9 vs. 21.1%, *p* = 0.62). The predicted *vs*. observed risk plots (Supplementary Figure 1) highlighted possible miscalibration, in men especially (HL-*p* = 0.09): the overall HL test based on the default of 10 groups (deciles) resulted in a good fit (*p* = 0.20), but the test was proven to be highly sensitive to the choice of group number (33), as indicated by HL-*p* values close to the significance level (*p* < 0.075) for the group numbers from 5 to 9 and for a few higher group numbers. The overall effect of the MRT predictors was significantly reduced in our cohort (*p* = 0.003), and in particular, the effect of sex was significantly smaller in the PROMISE cohort than that in the SMARTool population (*p* = 0.001). These results indicated that the model required updating. Among the MRT variables, only age, sex, smoking, diabetes, and hypertension were retained by the backward selection procedure (Table 3). The re-estimated model (reMRT) demonstrated an improved and more stable overall goodness of fit (*p* = 0.14) in men as well (*p* = 0.57), while the fit was worse in women (*p* = 0.02). The discrimination capability of reMRT was slightly higher (0.8463 vs. 0.8327, *p* = 0.31; 95% De Long confidence interval = 0.7856–0.9071) than that of the MRT, but the difference was not significant (see the comparison of the two ROC curves in Figure 1).

**Table 3:** Updated minimal risk tool: estimated coefficients.

**Variable**	**Coeff**.	**SE**	***p*^**a**^**
Intercept	2.625	1.720	0.127
Age	−0.120	0.028	≪0.001
Female sex	2.396	0.412	≪0.001
Never smoking	1.037	0.609	0.089
No diabetes	1.116	0.567	0.049
No hypertension	0.685	0.388	0.078

a *p≪0.001 means order of magnitude <-4*.

To better understand the effect of re-estimation, changes in risk classification due to updating have been described by the net reclassification index (NRI) at event rate (34). Table 4 shows that the reMRT improved the correct classification of CAD patients (NRI– = 0.117, lower limit = 0.06, upper limit = 0.17), while it slightly worsened the classification of normal subjects (NRI+ = −0.098, lower limit = −0.20, upper limit = 0). The total NRI(*p*) at *p* = 19.9% was 0.019 (lower limit = −0.09, upper limit = 0.13).

**Table 4:** Reclassification tables between the minimal risk tool (MRT) original model and the re-estimated model (reMRT) on the SMARTOOL cohort.

**Normal subjects**		**reMRT**		
		**CAD**	**Normal**	**Total**
**MRT**	**CAD**	7	1	8
	**Normal**	6	37	43
	**Total**	13	38	51
**CAD subjects**		**reMRT**		
		**CAD**	**Normal**	**Total**
**MRT**	**CAD**	131	5	136
	**Normal**	29	40	69
	**Total**	160	45	205

*NRI+ = −0.098, NRI– = 0.117, NRI = 0.019*.

*CAD, coronary artery disease; NRI, net reclassification index*.

### LRT Analysis: Predictive Improvement of Lipids

Except for SM(34:1) with only 226 values, the LRT was carried out on a subset of at least 240 subjects for each lipid (see Table 2). The probability of being at minimal risk was significantly better estimated by adding either Cer(d18:1/16:0) (*p* = 0.01), SM(40:2) (*p* = 0.04), or SM(41:1) at a lesser extent (*p* = 0.052) to reMRT than by applying the reMRT alone. The sensitivity analysis confirmed this result, as shown in Table 5. In fact, both the basic model and the basic-hsTnT model were suitable for the sensitivity analysis due to a good fit to the data (HL-*p*>0.50) and a high discrimination capability (AUC values of 0.8323 and 0.8807, respectively), as shown in Supplementary Table 1. Moreover, a few additional contributions were identified. Compared to reMRT, the prediction capabilities of the basic model were also enhanced by SM(34:1) (*p* = 0.003) and SM(41:2) (*p* = 0.03). Finally, compared to the previous two baseline models, the prediction capabilities of the basic-hs-cTnT model were also improved by SM(38:2) and SM(42:2) (*p* = 0.03 in both cases).

**Table 5:** Significance of the incremental values of selected lipids over the re-estimated minimal risk tool (reMRT) and the two alternative baseline models for the purpose of sensitivity analysis.

**Lipid species**	**LRT-p value**		
	**reMRT**	**Basic model**	**Basic-hs-cTnT model**
Cer(d18:1/16:0)	**0.01**	**0.01**	**0.02**
TG(52:2)	0.41	0.36	0.89
TG(52:3)	0.49	0.54	0.83
TG(54:2)	0.76	0.25	0.92
TG(54:3)	0.50	0.33	0.57
SM(34:1)	0.12	**0.003**	**0.01**
SM(36:2)	0.30	0.34	0.17
SM(38:2)	0.16	0.09	**0.03**
SM(40:3)	0.88	0.15	0.12
SM(40:2)	**0.04**	**0.006**	**0.001**
SM(41:2)	0.18	**0.03**	**0.01**
SM(41:1)	**0.052**	**0.054**	**0.005**
SM(42:4)	0.77	0.17	**0.03**
SM(42:3)	0.76	0.24	0.10

*The p-value of the likelihood ratio test (LRT) is reported for the reMRT model, including age, sex, smoking, diabetes, and hypertension; the basic model, including age, sex, and symptoms; and the basic-hs-cTnT (high-sensitive cardiac troponin T) model, including age, sex, symptoms, and high-sensitive cardiac troponin T (hs-cTnT). Bold charcter highlight significant incremental values*.

As a further step, the lipids Cer(d18:1/16:0), SM(40:2), SM(34:1), SM(41:1), SM(41:2), SM(38:2), and SM(42:4) were simultaneously considered in a multivariable logistic model to evaluate their joint value for predictive improvement. The model was fitted on the 226 subjects with complete data on all these variables. With the backward selection method, SM(41:1) and SM(41:2) were discarded.

The selected model is summarized in Table 6. The enhanced linear predictor was then obtained by adding (on top) the linear predictor coming from Table 6 to the reMRT and the other baseline models. The LRT indicated a very strong predictive improvement when Cer(d18:1/16:0), SM(40:2), SM(34:1), SM(38:2), and SM(42:4) are jointly considered (*p* < 0.00001), regardless of the baseline model.

**Table 6:** Multivariable logistic regression estimated model.

**Variable**	**Coeff**.	**SE**	***p*-value^**a**^**
Intercept	−8.325	1.676	≪0.001
Cer(d18:1/16:0)	0.675	0.244	0.006
SM(34:1)	0.027	0.010	0.006
SM(38:2)	0.136	0.084	0.106
SM(40:2)	0.038	0.026	0.138
SM(42:4)	−0.196	0.112	0.080

*The model was estimated on 226 subjects*.

a *p≪0.001 means order of magnitude <-4*.

Table 5 shows that none of the differently expressed triglycerides brought significant added value, regardless of the baseline model (*p*≥0.25).

## Discussion

It is well-known that coronary artery disease is present only in a percentage of patients referred to diagnostic testing for chest pain and that as many as one-fourth of the CCTAs performed as the recommended first-line test are unnecessary. Therefore, the application in clinical practice of a predictive model able to directly exclude the majority of patients without coronary lesions from unnecessary diagnostic testing before referral is highly relevant to optimize patient management and for cost effectiveness (35–37). Minimizing unnecessary tests, procedures, and costs is an increasing need in the last years (4, 24, 38). The usually suggested way of ruling out CAD is based on a pretest probability of obstructive CAD lower than a given threshold, usually from 5 to 15%, for instance (1, 39, 40).

Such a rule-out strategy may not be completely efficient, as also recently demonstrated (41). When a model built to discriminate obstructive CAD like ESC-PTP is used to also discriminate the absence of atherosclerosis, this eventuality is part of the game, as also noted by Adamson et al. (5). Indeed, when predictive probability models are empirically derived, a set of variables proving to be good predictors of obstructive CAD can fail to discriminate subjects at minimal risk, and *vice versa* (42, 43). The major novelty introduced by the MRT is its being the first model proposing an endpoint directly aimed at identifying those subjects with normal arteries (3), and for this reason, we have chosen it as the baseline model for testing the added value of a few targeted lipids as novel and useful biomarkers to rule out CAD.

Several serum sphingolipids have been proposed as independent biomarkers of clinically manifest CAD. Lipid-based test scores, such as CERT2 (10), are widely recognized for their prognostic value for any type of cardiovascular event, and significant associations with non-calcified coronary artery plaque burden assessed by CCTA have been reported (44). Recently, it has been found that, in statin users, the CAD severity and atherosclerotic lipid burden assessed by CCTA are significantly associated with specific circulating lipid species among the classes of SMs and PEs (13). Sphingolipids play an important role in intracellular signal transduction and regulate cellular processes such as proliferation, maturation, and apoptosis; they are also involved in cellular stress responses. It is known that atherosclerosis can increase sphingomyelinase (SMase) activity, consequently altering ceramide and sphingomyelin plasma concentrations (45). Additionally, SMs are reported to be the second most abundant phospholipid component and the major sphingolipid in HDL particles (46), which play a crucial role in atheroprotection by driving reverse cholesterol transport, and lipid changes in HDL composition can reduce their cholesterol efflux capacity. In details, the beneficial role attributed to HDL-C actually is thought to reflect the multiple cardioprotective properties of HDL particles, which primarily include its capacity to efflux cholesterol from peripheral cells (reverse cholesterol transport, RCT), but may also involve antioxidative, anti-inflammatory, anti-apoptotic, anti-thrombotic, anti-infectious, and anti-diabetic activities (47). Nevertheless, conflicting evidences are recently emerging on the protective role of HDL-C in cardiovascular health. Observations of hereditary syndromes featuring scant HDL-C in the absence of premature atherosclerotic disease and very high levels of this lipoprotein that do not appear to grant additional benefits indicate that HDL-C level *per se* may not be a good predictor of cardiovascular disease. Indeed, current knowledge suggests that the biological activity of HDL may not depend solely on its concentration but also on its quality, as alterations in various structural components lead to a state of dysfunction independently of their serum concentrations (48). In this context, the cholesterol efflux capacity of HDL appears a more effective feature in predicting cardiovascular disease than does the HDL-C level. SM species are reported to be one of the most abundant lipid components in HDL particles and, according to their surface distributions, play a key role on cholesterol efflux capacity. Dysfunctional HDL exhibits 25% less lipids per milligram of protein, reflecting lower contents of SM and PC, and a substitution of 50% of cholesteryl ester for TG (49). These lipid changes can alter the anti-atherogenic HDL assets, reducing their cholesterol efflux capacity and hindering RCT. Accordingly, from a pathophysiological point of view, the dysregulation of particular lipid species might reflect the functionality of HDLs better than the overall HDL-C plasma level.

The results of the present proof-of-concept study extend the clinical relevance of all these previous evidences by demonstrating that the plasma concentrations of some plasma sphingolipids, which are significantly associated with coronary atherosclerosis *per se*, provide an increased predictive value when added to the MRT model for ruling out CAD.

We validated the MRT on a small population with a low prevalence of subjects at minimal risk. In principle, the MRT is an appropriate pretest stratification model also for our population, where no links between patient-reported symptom presentation and obstructive CAD is observed, in agreement with previous studies (50–52). At a first analysis, the model has shown calibration in-the-large on the SMARTool cohort and a good fit. A more in-depth validation procedure, however, allowed us to gain deeper insights into the need for a model re-estimation, essentially due to a significantly higher effect of the gender variable in our cohort than that in the PROMISE cohort. The re-estimated model, reMRT, did not include the basic lipid profile of the patients (dyslipidemia and HDL) or a family history of CAD in the selected predictors. From a methodological point of view, a comparison with the validations carried out on the SCOT-HEART (5) and the Dan-NICAD (6) populations is not possible. In the former case, erroneous coefficients were used (4, 52); in the latter, a different validation procedure based on calibration plots was applied.

Then, the reMRT was used as a baseline model to assess the improvements provided by each of a few targeted lipids in the prediction of subjects at minimal risk. Among the considered outcome-associated lipids, one ceramide, Cer(d18:1/16:0), and two sphingomyelins, SM(40:2) and SM(41:1), were able to add predictive value to the model in ruling out coronary atherosclerosis.

The sensitivity analysis confirmed the results obtained in reMRT: Cer(d18:1/16:0), SM(40:2), and SM(41:1) added significant value to the other two different models as well, slightly weaker in the case of SM(41:1) only.

In addition, when the basic-hs-cTnT model is considered as a baseline model, also SM(34:1), SM(38:2), SM(41:2), and SM(42:4) added a significant value. This finding supports the potential additive predictive value of SMs also in combination with a novel sensitive biomarker such as high-sensitive troponin T (hs-cTnT), currently regarded as a marker of not only myocardial injury in acute coronary syndrome but also of CAD severity in stable ischemic heart disease (53). In our analysis, hs-cTnT, which had a strong association with the normal condition in our study population (*p* ≪ 0.001), is in turn able to significantly increase the probability of excluding the presence of CAD when added to the reMRT model (*p* = 0.008). Moreover, when we limited to the subpopulation of 225 subjects with complete data on all the variables, the basic-hs-cTnT model showed the highest discrimination capability among the three baseline models [0.8807 *vs*. 0.8467 (reMRT) and 0.8392 (basic), DeLong 95% confidence interval = 0.8288–0.9327], albeit not statistically significant when compared to reMRT (*p* = 0.20), as expected due to the high AUC values and small sample size (54).

The major limitation of this study is the low absolute number of subjects in the two groups (normal and CAD) undergoing plasma lipidomics and CCTA. Moreover, the lipid and hs-cTnT concentrations were not available in about one-tenth of patients, further reducing the study cohort in part of the analysis. Finally, an unbalance in gender, with a low number of females in the overall cohort (37.5%) due to an enrollment strategy focused on suspected coronary artery disease and indication to perform CCTA and a strong prevalence (72.5%) of females in the group of normal subjects, must be acknowledged. Although this limit has been partially addressed by recalibration and re-estimation of the MRT model in our cohort, we cannot completely exclude a gender effect on the main findings of our study. This would represent a major concern when developing a clinical PTP model based on the current results: a larger and more gender-balanced derivation and validation population would then be necessary, as already planned for future development.

## Conclusions

We found that the plasma concentrations of specific sphingolipids [Cer(d18:1/16:0), SM(40:2), and SM(41:1)] can improve the accuracy of pretest stratification of suspected CAD patients referred to CCTA when added to the MRT model. The correct identification of these subjects, who derive minimal benefit and value from diagnostic tests such as the CCTA, meets the clinical need of a more cost-efficient use of diagnostic imaging with a reduction of unnecessary radiation exposure for subjects and operators. The results of our proof-of-concept study support the future exploitation of plasma lipidomics-derived biomarkers in clinical practice, not only to improve the prediction of obstructive CAD, vulnerable plaques, or long-term adverse cardiovascular outcomes but also to help ruling out coronary atherosclerosis in patients referred to CCTA as the first-line test for suspected CAD.

## Data Availability Statement

The original contributions presented in the study are included in the article/Supplementary Material, further inquiries can be directed to the corresponding author/s.

## Ethics Statement

The studies involving human participants were reviewed and approved by Ethical Commitee of Area Vasta Nord-Ovest (Italy). The patients/participants provided their written informed consent to participate in this study.

## Author Contributions

AB designed the statistical approach, conducted the statistical analysis, and wrote the original draft of the manuscript. EM, ND, CC, and GS conducted the experimental investigation on lipidomics and biohumoral data and helped review the manuscript. DN, JS, and AS conducted the patient enrollment and CCTA image analysis. PM and CL provided feedback on statistical analysis and methodology and helped write and review the manuscript. GP collected and organized all data from clinical to CCTA and lipidomics and wrote and reviewed the manuscript. SR wrote and reviewed the manuscript, managed the project, and obtained research funding. All authors have read and agreed to the published version of the manuscript.

## Conflict of Interest

The authors declare that the research was conducted in the absence of any commercial or financial relationships that could be construed as a potential conflict of interest.
