# Supplementary material for: Predictive Added Value of Selected Plasma Lipids to a Re-estimated Minimal Risk Tool
Source: Front Cardiovasc Med. 2021 Jul 16;8:682785. doi: 10.3389/fcvm.2021.682785 (PMC8322727; doi:10.3389/fcvm.2021.682785)
Supplement: Supplementary file 1 [file Data_Sheet_1.docx]

Supplementary Material

# Supplementary Figures and Tables

## Supplementary Table

## Supplementary Table 1 - Estimated baseline models: estimated parameters and their significance, $\mathbf{p}$-value of the Hosmer-Lemeshow goodness-of-fit test (HL-$\mathbf{p}$), AUCs and their 95% confidence intervals.

| **Model** |  |  |  |  |  |  |  | |
| --- | --- | --- | --- | --- | --- | --- | --- | --- |
|  | **Variable** | **Coeff.** | **Std. Err.** | $\boldsymbol{p}$**^1^** | **HL-**$\boldsymbol{p}$ | **AUC** | **95% CI^2^** | |
|  |  |  |  |  |  |  |  |  |
| **Basic Model** |  |  |  |  | 0.87 | 0.8323 | 0.7728-0.8919 | |
|  | Intercept | 5.271 | 1.548 | $<0.001$ |  |  |  | |
|  | Age | -0.118 | 0.026 | $\ll0.001$ |  |  |  | |
|  | Female sex | 2.277 | 0.410 | $\ll0.001$ |  |  |  | |
|  | Chest pain  (Reference group: Typical) |  |  |  |  |  |  | |
|  | Atypical | -0.605 | 0.423 | 0.152 |  |  |  | |
|  | Non-anginal | -1.314 | 0.533 | 0.014 |  |  |  | |
|  |  |  |  |  |  |  |  |  |
| **basic-hsTnT**  **model^3^** |  |  |  |  | 0.56 | 0.8807 | 0.8288-0.9327 | |
|  | Intercept | 7.571 | 2.020 | $<0.001$ |  |  |  | |
|  | Age | -0.123 | 0.033 | $<0.001$ |  |  |  | |
|  | Female sex | 2.022 | 0.0466 | $\ll0.001$ |  |  |  | |
|  | Chest pain  (Reference group: Typical) |  |  |  |  |  |  | |
|  | Atypical | -0.585 | 0.490 | 0.232 |  |  |  | |
|  | Non-anginal | -1.832 | 0.637 | 0.004 |  |  |  | |
|  | hs-cTnT | -0.322 | 0.096 | $<0.001$ |  |  |  | |
| ^1^ $\ll0.001$means order of magnitude less than -4.  ^2^ DeLong method.  ^3^ The model has been estimated on the 225 subjects with complete data on hs-cTnT | | | | | | | |  |

## Supplementary Figure

**Supplementary Figure 1.** **Calibration plot of the original MRT on the SMARTool cohort: Predicted probability of minimal risk vs. the observed frequency of minimal risk. Black circles are for men and gray circles for women. The number of patients per decile category are reported.**
